# Long-Term Outcomes of Minimally Invasive vs. Traditional Open Spinal Fusion: A Comparative Analysis

**Published:** 2025-03-26

**Authors:** Bahram Saber, Devendra K. Agrawal

**Affiliations:** 1 Department of Translational Research, College of Osteopathic Medicine of the Pacific, Western University of Health Sciences, Pomona CA 91766, USA.

**Keywords:** Adjacent Segment Disease (ASD), Cost-Effectiveness in Spine Surgery, Fusion Success Rates, Healthcare Utilization in Spine Surgery, Lateral lumbar interbody fusion, Long-Term Spine Surgery Outcomes, Minimally Invasive Spinal Fusion (MISF), MISF vs. Open TLIF, Open Spinal Fusion, Pain Relief in Spinal Fusion, Perioperative Complications, Postoperative Recovery, Surgical Biomechanics, Spinal Degenerative Disease Treatment, Spinal Instrumentation, Spinal Surgery Outcomes, Traditional open spinal fusion, Transforaminal lumbar interbody fusion (TLIF)

## Abstract

Spinal fusion is a widely performed surgical intervention for managing degenerative spinal conditions, instability, and deformities. Traditionally, open spinal fusion has been the standard approach, offering direct visualization and access to spinal structures. However, advancements in surgical techniques have led to the development of minimally invasive spinal fusion (MISF) as an alternative, aiming to achieve comparable clinical outcomes while reducing surgical trauma, postoperative pain, and recovery time. Despite these advantages, concerns remain regarding the long-term effectiveness of MISF, particularly in terms of fusion rates, complication risks, and adjacent segment disease (ASD). This review critically examines the long-term outcomes of MISF compared to traditional open fusion, focusing on key factors such as perioperative outcomes, pain relief, functional recovery, fusion success rates, and cost-effectiveness. Perioperative data indicate that MISF is associated with reduced blood loss, shorter hospital stays, and lower infection rates but may involve longer surgical times and a steeper learning curve. Long-term clinical outcomes appear comparable between MISF and open fusion, with both techniques achieving high fusion rates and significant improvements in pain and function. However, the impact of MISF on adjacent segment disease remains inconclusive, with conflicting evidence regarding its potential biomechanical advantages. Cost-effectiveness analyses suggest that MISF may offer financial advantages in the long term by reducing hospitalization and rehabilitation expenses, despite higher initial surgical costs. Nonetheless, limitations in current research, including variability in study methodologies, patient selection, and surgeon expertise, necessitate further high-quality, long-term randomized controlled trials. This review synthesizes the current literature on MISF and traditional open fusion, identifies existing research gaps, and outlines future directions for optimizing surgical decision-making and improving patient outcomes.

## Introduction

Spinal fusion is a widely performed surgical procedure for treating degenerative spinal conditions, instability, and deformities [1, 2]. Traditionally, open spinal fusion has been the standard approach, providing direct visualization and access to the spinal structures [3]. However, advances in surgical techniques have led to the development of minimally invasive spinal fusion (MISF) as an alternative. MISF aims to achieve the same clinical outcomes as open fusion while reducing surgical trauma, postoperative pain, potential neurological complications, and recovery time [4, 5].

Minimally invasive techniques utilize smaller incisions, specialized retractors, and percutaneous instrumentation to minimize disruption to surrounding tissues. This approach has been associated with reduced blood loss, shorter hospital stays, and faster rehabilitation compared to traditional open fusion [4]. Despite these advantages, concerns remain regarding the long-term effectiveness of MISF, including fusion rates, complication risks, and the development of adjacent segment disease [6].

As the adoption of MISF continues to grow, it is essential to evaluate its long-term outcomes compared to traditional open techniques. This review aims to examine the available literature on both surgical approaches, analyzing their impact on patient outcomes, complication rates, and overall efficacy. By synthesizing current evidence, this review will provide insight into the potential benefits and limitations of MISF and its role in modern spinal surgery.

### Surgical Techniques and Biomechanical Considerations

#### Minimally Invasive Spinal Fusion (MISF) Approach

Minimally invasive spinal fusion (MISF) is designed to reduce surgical trauma by limiting soft tissue disruption and preserving paraspinal musculature [7]. The procedure typically involves small incisions, tubular retractors, and percutaneous pedicle screw placement. Fluoroscopy or intraoperative navigation assists in guiding instrumentation with greater precision, minimizing damage to surrounding structures. Common MISF techniques include minimally invasive transforaminal lumbar interbody fusion (MI-TLIF) and lateral lumbar interbody fusion (LLIF), both of which aim to achieve spinal stability while reducing operative morbidity [7].

Compared to traditional open fusion, MISF is associated with reduced intraoperative blood loss, lower rates of infection, and faster postoperative recovery [8]. However, the steep learning curve, longer operative times, and potential for increased radiation exposure to the surgical team remain key considerations. Additionally, the limited surgical exposure in MISF can make achieving adequate decompression and optimal fusion rates more challenging [8].

#### Traditional Open Spinal Fusion

Open spinal fusion has long been considered the gold standard for treating spinal instability and degenerative conditions [9]. This approach involves a midline incision, muscle dissection, and direct visualization of the spinal anatomy. Traditional open techniques allow for extensive decompression of neural elements and precise placement of interbody grafts and pedicle screws. The increased exposure provides surgeons with greater control over bony preparation and implant positioning, potentially leading to higher fusion rates [9].

Despite its advantages, open spinal fusion is associated with increased perioperative morbidity, including higher rates of blood loss, longer hospital stays, and greater postoperative pain [10]. The extensive muscle dissection required for open fusion can also contribute to paraspinal muscle atrophy, which may impact long-term spinal function. These factors have driven the development and adoption of MISF as a viable alternative [10].

#### Biomechanical and Structural Considerations

Both MISF and open fusion aim to achieve spinal stability and solid arthrodesis. The biomechanical properties of each approach differ due to variations in surgical exposure and instrumentation techniques. Studies have suggested that MISF may reduce the incidence of adjacent segment disease (ASD) by preserving posterior musculature and ligamentous structures, which play a role in distributing biomechanical stress [11]. However, the potential for lower fusion rates in MISF compared to open techniques remains a topic of ongoing research.

Traditional open fusion, while effective in achieving strong bony fusion, can alter spinal biomechanics by increasing stress on adjacent segments [12]. This may contribute to a higher incidence of ASD over time. Additionally, differences in cage placement, bone grafting techniques, and fixation methods between the two approaches may influence long-term stability and clinical outcomes [12].

As research continues to evolve, understanding the biomechanical implications of each technique is critical in optimizing patient selection and surgical planning. Future studies focusing on long-term follow-up and comparative analyses will help refine the role of MISF and open fusion in spinal surgery.

#### Perioperative Outcomes and Complications

Minimally invasive spinal fusion (MISF) has gained popularity due to its potential advantages in perioperative outcomes compared to traditional open fusion techniques. Several key factors, including surgical duration, blood loss, length of hospital stay, and complication rates, play a crucial role in determining the overall effectiveness and safety of these approaches.

#### Surgical Time

One of the notable differences between MISF and open fusion is the duration of surgery. While MISF often requires longer operative times due to the specialized techniques and intraoperative imaging necessary for precision, this is offset by its reduced impact on soft tissues [13]. Traditional open fusion, in contrast, allows for a broader surgical field, which may expedite the placement of instrumentation but at the cost of increased tissue disruption [14].

#### Blood Loss and Transfusion Rates

**Figure 1:**
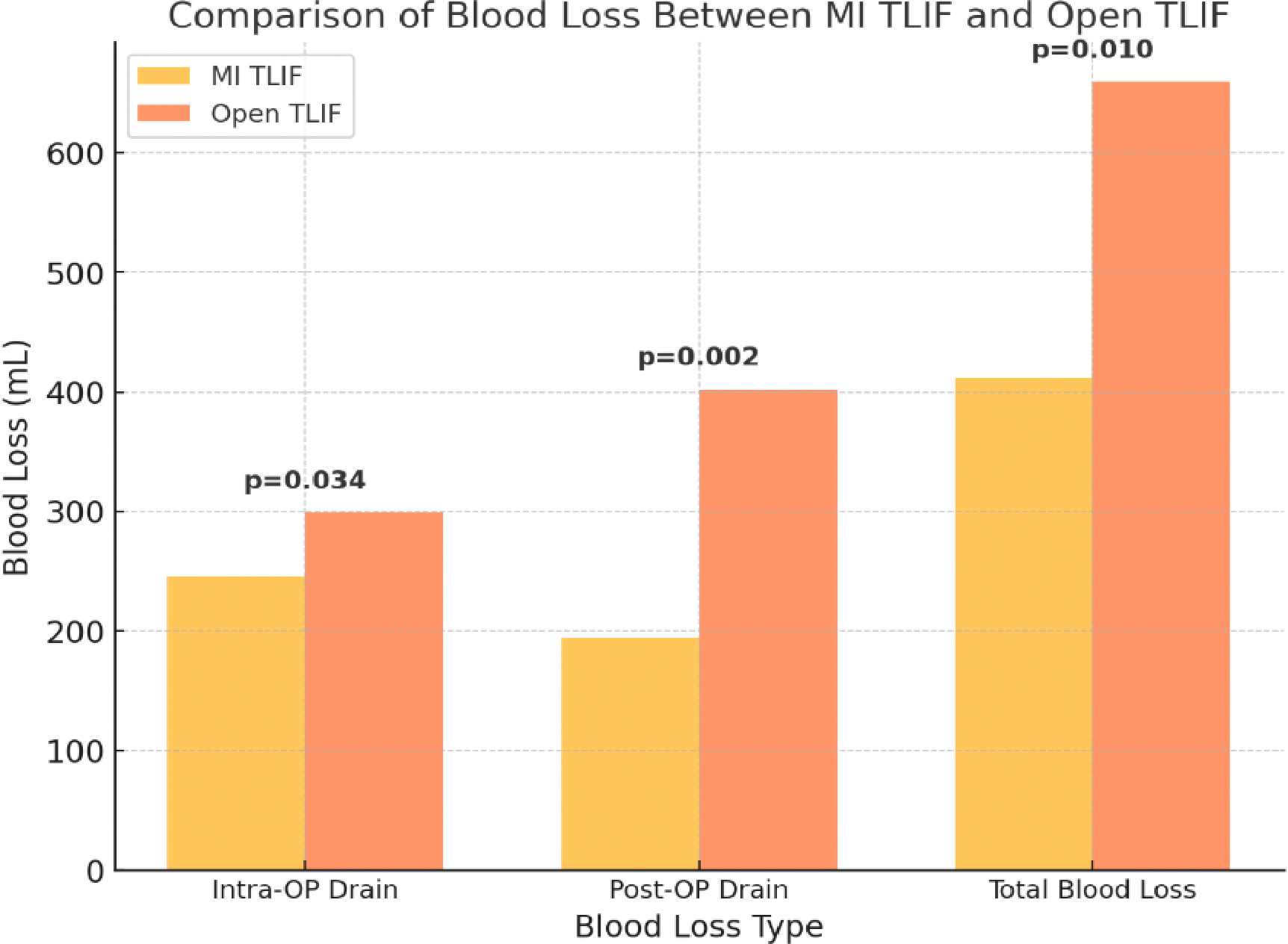
Comparison of intraoperative drain, postoperative drain, and total blood loss between minimally invasive transforaminal lumbar interbody fusion (MI-TLIF) and open TLIF, (N=97). Data compiled and redrawn from the findings in Hong et al [15].

MISF is associated with significantly lower intraoperative blood loss compared to open fusion [15]. Studies have shown that the muscle-preserving approach in MISF leads to reduced bleeding, thereby lowering the likelihood of requiring blood transfusions. In contrast, open fusion techniques involve greater soft tissue dissection, which increases blood loss and may necessitate transfusion in a higher percentage of cases [15]. As seen in figure 1, researchers found that Open TLIF demonstrated significantly higher blood loss in intraoperative drain, postoperative drain, and total blood loss.

#### Hospital Length of Stay

**Figure 2:**
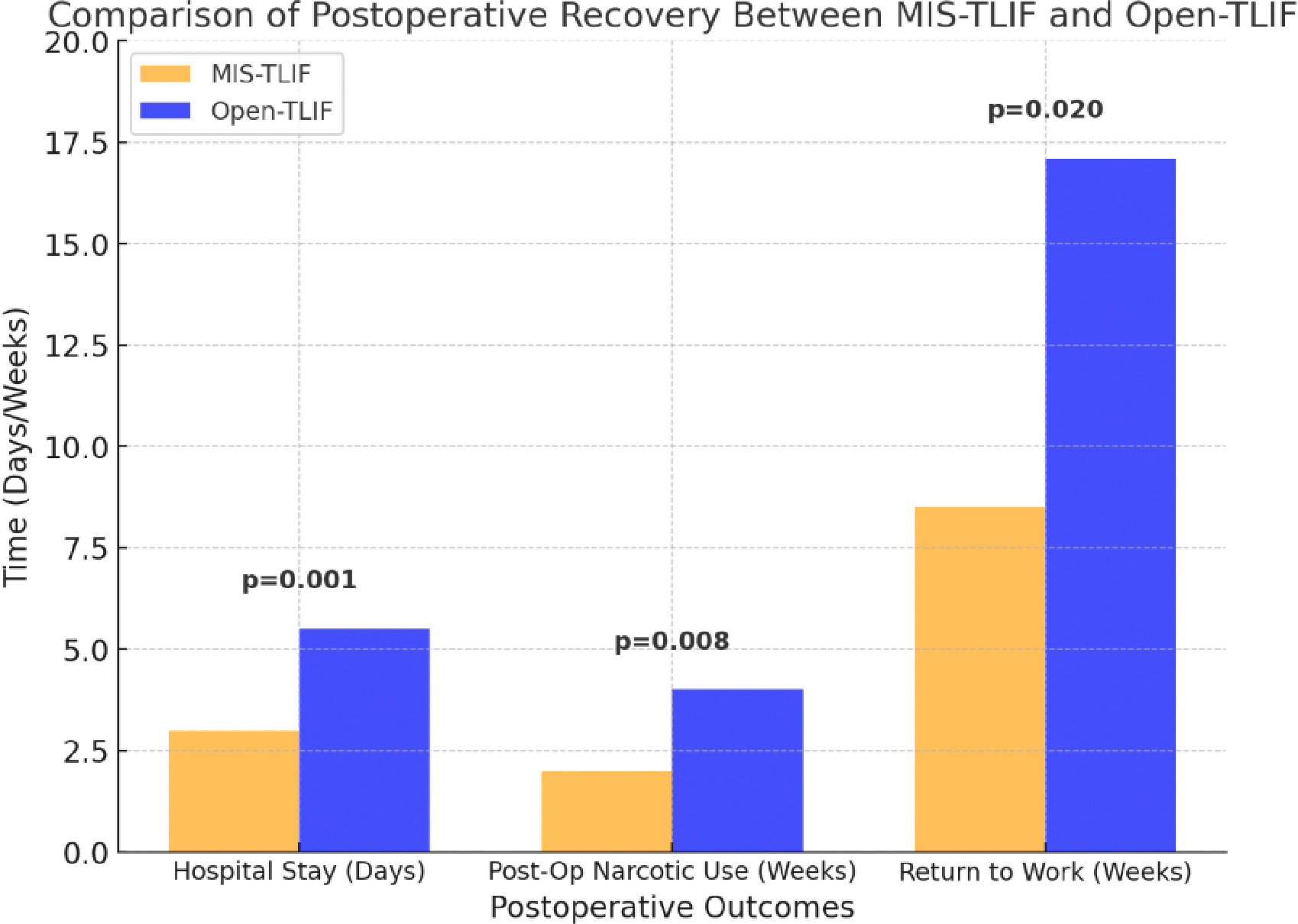
Comparison of postoperative recovery metrics between MIS-TLIF and Open-TLIF, including median hospital stay duration, postoperative narcotic use, and time to return to work, (N = 30). Data compiled and redrawn from the findings in Adogwa et al. (16).

The minimally invasive approach has been linked to shorter hospital stays due to reduced postoperative pain and quicker mobilization [16]. Patients undergoing MISF typically require less postoperative opioid medication and demonstrate faster recovery timelines. Open fusion, however, often results in longer hospitalization due to the increased need for pain management and rehabilitation [16]. As seen in figure 2, patients undergoing MIS-TLIF experienced significantly shorter hospital stays, shorter duration of post-op narcotic use, and quicker return to work [16].

#### Complication Rates

Despite its advantages, MISF is not without risks. The steep learning curve for surgeons, along with the reliance on intraoperative fluoroscopy or navigation, may contribute to technical challenges such as screw misplacement [17]. However, research suggests that MISF has lower rates of surgical site infections due to smaller incisions and less soft tissue disruption. Open fusion, while allowing for more direct visualization, carries higher risks of infection, postoperative pain, and adjacent segment degeneration due to increased paraspinal muscle damage [18].

Overall, while MISF presents numerous perioperative benefits, it is essential to weigh these against potential challenges such as longer surgical times and the need for advanced training. The decision between MISF and open fusion should be tailored to the patient’s condition, surgeon expertise, and long-term surgical goals.

#### Long-Term Clinical and Functional Outcomes

Evaluating the long-term outcomes of minimally invasive spinal fusion (MISF) versus traditional open fusion is crucial in determining their respective effectiveness in treating spinal disorders. Key measures of success include pain relief, functional recovery, fusion success rates, and the development of adjacent segment disease (ASD) [19].

#### Pain Relief

**Figure 3:**
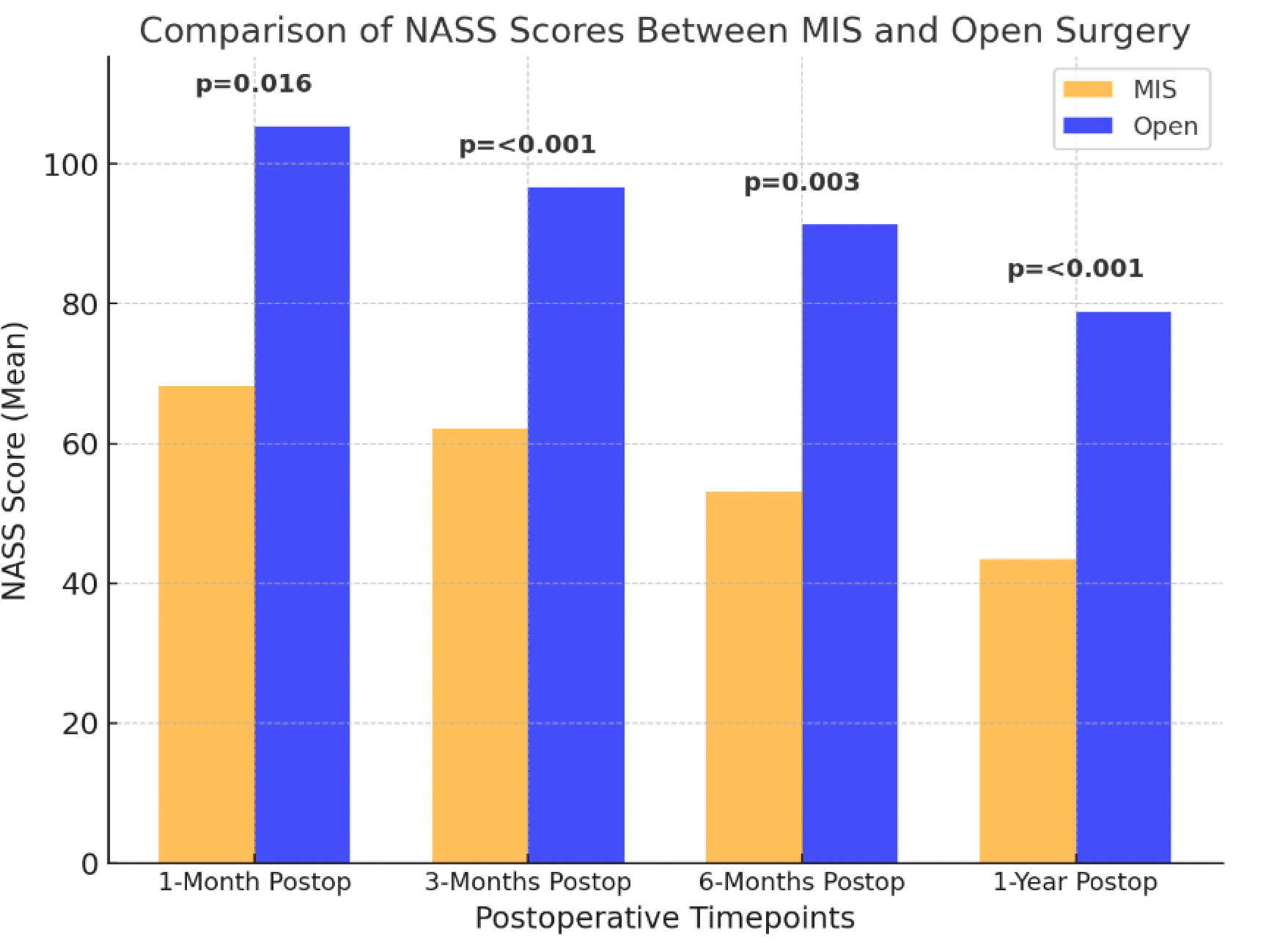
North American Spine Society (NASS) scores between MIS and open surgery at postoperative time points, (N=129). Data compiled and redrawn from the findings in Lee et al [19].

One of the primary objectives of spinal fusion is to reduce chronic pain associated with degenerative spine conditions. North American Spine Society (NAAS) low back pain scores are commonly used to assess pain post-operatively [19]. The North American Spine Society (NASS) score evaluates spinal function and patient-reported outcomes related to pain and mobility. Lower scores were known to have better outcomes. Figure 3 displays North American Spine Society (NASS) scores between MIS and open surgery at postoperative time points where statistically significant differences were observed [19]. MIS demonstrated consistently lower NASS scores, suggesting improved clinical outcomes.

#### Functional Recovery

**Figure 4:**
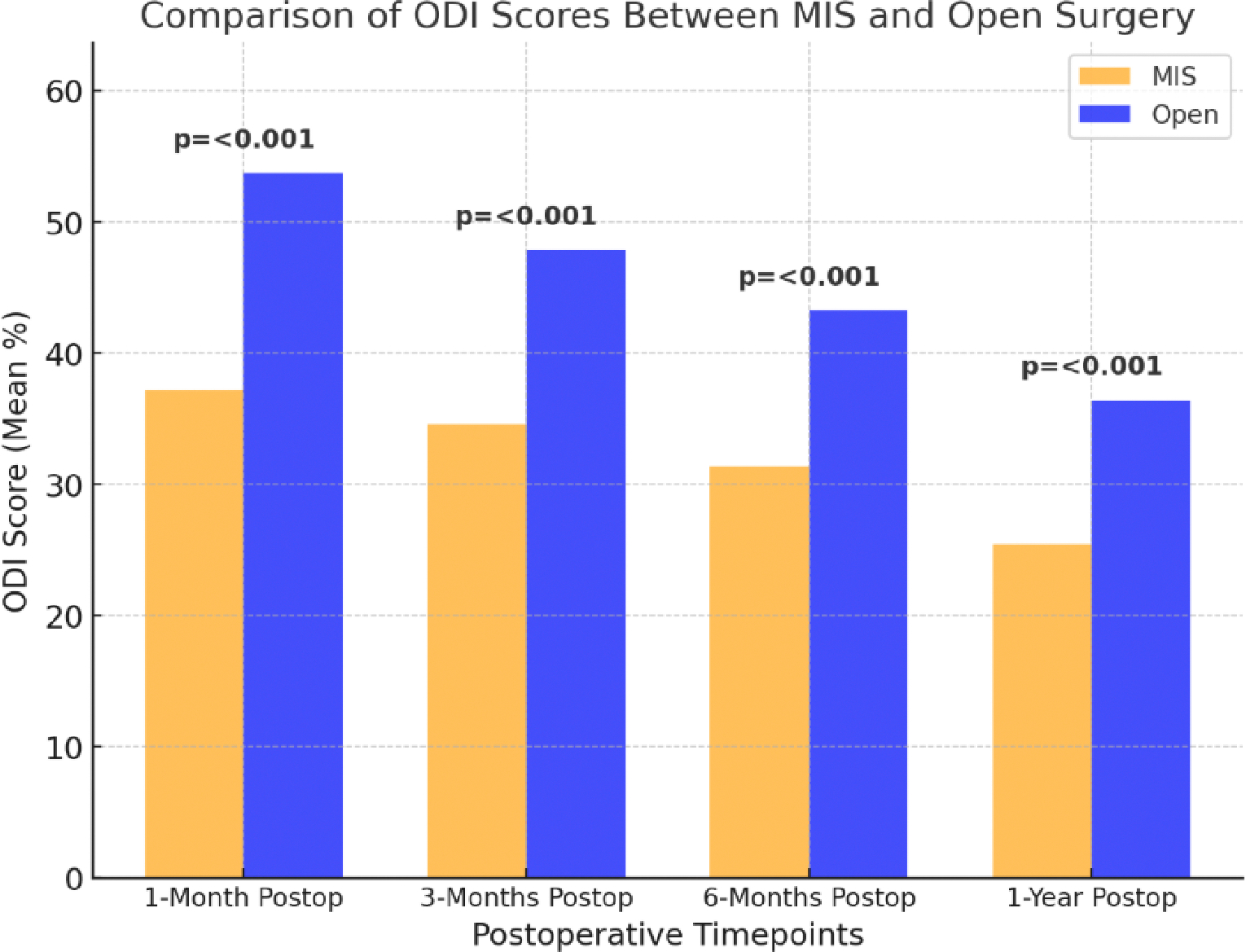
Differences in Oswestry Disability Index (ODI) scores at statistically significant postoperative time points between minimally invasive spine surgery (MIS) and open surgery, (N=129). Data compiled and redrawn from the findings in Lee et al [19].

Return to daily activities, mobility, and work status are critical indicators of surgical success. The Oswestry Disability Index (ODI) score is used to assess disability improvement postoperatively. It assesses the degree of disability and functional impairment caused by lower back pain and lower scores were known to have better outcomes. Figure 4 illustrates the differences in Oswestry Disability Index (ODI) scores at statistically significant postoperative time points between minimally invasive spine surgery (MIS) and open surgery [19]. MIS consistently resulted in lower ODI scores, indicating better functional recovery [19]. MISF is often associated with a faster return to normal function due to its less invasive nature, lower postoperative pain levels, and quicker rehabilitation times [20]. In contrast, open fusion may lead to prolonged recovery times, particularly due to extensive muscle dissection and higher postoperative pain [20].

#### Fusion Success Rates

The primary goal of spinal fusion is to achieve solid arthrodesis between vertebral segments, preventing motion at the affected spinal level [21]. Radiographic studies indicate that fusion rates are similar between MISF and open fusion, with both methods demonstrating fusion rates above 90% in long-term follow-ups [21].

#### Adjacent Segment Disease (ASD)

One of the most debated long-term concerns in spinal fusion is adjacent segment disease (ASD)—the degeneration of spinal levels adjacent to the fused segment. Some studies suggest that MISF may reduce the risk of ASD due to its muscle-sparing approach, which helps maintain segmental mobility [22]. In contrast, open fusion may contribute to higher ASD rates due to increased paraspinal muscle disruption, which can alter biomechanical loading [23]. However, other studies indicate no significant difference in ASD rates between the two techniques over a long-term follow-up period [24].

Overall, while MISF provides several short-term advantages, long-term clinical and functional outcomes appear to be comparable to open fusion in terms of pain relief, functional improvement, and fusion success rates. The impact on adjacent segment disease remains a topic of ongoing research, with mixed findings regarding whether MISF offers a protective effect.

#### Cost-Effectiveness and Healthcare Utilization

The economic burden of spinal fusion surgery is a critical factor in determining its overall value for both patients and healthcare systems. Comparing minimally invasive spinal fusion (MISF) and traditional open fusion involves assessing hospital costs, length of stay, reoperation rates, and long-term financial implications.

#### Hospital Costs

Minimally invasive spinal fusion (MISF) procedures often incur higher initial surgical costs due to specialized instrumentation, intraoperative navigation, and the need for surgeon training [25]. However, these upfront expenses may be offset by benefits such as shorter hospital stays, reduced postoperative complications, and faster recovery times, potentially leading to lower total hospital costs over time. For example, one study found that MIS techniques resulted in a statistically significant cost savings of $2,825.37 (10.4%) compared to traditional open techniques, primarily due to decreased hospital operating costs and fewer complications [25]. Additionally, another study found reduced costs associated with MIS compared to open surgery, particularly in single- and two-level transforaminal lumbar interbody fusion (TLIF) procedures, suggesting better cost-effectiveness for MISF [26]. These findings indicate that while MISF may have higher initial costs, the overall healthcare expenditure could be lower due to reduced postoperative complications and rehabilitation expenses.

#### Patient Satisfaction and Quality of Life

A key measure of healthcare value is patient-reported satisfaction and quality of life following spinal fusion. Patients undergoing MISF generally report higher satisfaction rates in the early postoperative period due to less pain, faster recovery, and reduced hospital stays [27]. However, at long-term follow-up (5–10 years), satisfaction scores between MISF and open fusion tend to equalize, with both techniques achieving similar functional improvements and pain relief [28].

#### Long-Term Financial Implications

The long-term financial impact of spinal fusion extends beyond the initial surgery. Factors such as lost wages due to prolonged recovery, need for rehabilitation, and potential for future spine procedures all influence the overall cost-effectiveness of the procedure. The ability of MISF to accelerate return to work and reduce hospital-related expenses may provide a greater cost-benefit ratio in the long term and may play a crucial role in making such procedures more economically viable for our aging population [29].

## Discussion

The debate between minimally invasive spinal fusion (MISF) and traditional open fusion remains a focal point in spinal surgery research. While MISF offers perioperative advantages such as reduced blood loss, shorter hospital stays, and quicker recovery, the long-term clinical outcomes appear comparable to open fusion. This section will analyze the key findings from previous sections, highlight strengths and limitations in current research, and discuss potential future directions.

### Summary of the Findings

- Perioperative Benefits of MISF:
  - Lower intraoperative blood loss
  - Shorter hospital stays and faster early recovery
  - Reduced postoperative pain and infection risk
- Long-Term Outcomes:
  - Similar pain relief and functional improvements as open fusion
  - Comparable fusion success rates with advances in MISF techniques
  - Mixed evidence on the impact of MISF on adjacent segment disease (ASD)
- Cost-Effectiveness Considerations:
  - Higher initial costs for MISF due to specialized technology
  - Potential for lower total healthcare costs due to fewer complications
  - Faster return to work may favor MISF in long-term financial impact

### Limitations in Current Research

While numerous studies have compared MISF and open fusion, several gaps remain in the literature:

- **Lack of high-quality, long-term randomized controlled trials (RCTs)** comparing outcomes beyond 10 years.
- **Variability in patient populations** – many studies focus on single-level fusions, making it difficult to generalize to multi-level disease.
- **Heterogeneity in surgical techniques and instrumentation**, leading to inconsistent findings across studies.
- **Surgeon expertise bias** – outcomes may vary significantly depending on a surgeon’s experience with MISF techniques.

### Future Research Directions

To better understand the true long-term impact of MISF vs. open fusion, future studies should focus on:

1. **Large-scale RCTs with long-term follow-up (10+ years)** to evaluate functional outcomes and reoperation rates.
2. **Comparative studies on ASD progression**, particularly examining whether MISF offers a protective advantage.
3. **Cost-effectiveness analyses incorporating indirect costs**, such as time off work and long-term disability rates.
4. **Advancements in MISF technology**, including robotic-assisted fusion and patient-specific implants, to assess their role in improving outcomes.

Overall, while MISF provides clear short-term benefits, the long-term outcomes remain largely comparable to open fusion. The decision between these techniques should be based on patient-specific factors, surgeon expertise, and the complexity of the spinal pathology. Continued research is essential to further refine surgical techniques and optimize patient outcomes.

## Major Key Points

### 1. Surgical Techniques and Biomechanics

- MISF minimizes muscle disruption by using small incisions, tubular retractors, and percutaneous pedicle screw placement, reducing perioperative morbidity.
- Traditional open fusion provides direct visualization and greater control over implant positioning but at the cost of increased soft tissue damage.
- Biomechanical considerations suggest that MISF may better preserve posterior musculature, potentially reducing the incidence of adjacent segment disease (ASD), though this remains debated.

### 2. Perioperative Outcomes and Complications

- MISF has lower intraoperative blood loss and shorter hospital stays, making it advantageous for early recovery.
- Surgical time is often longer for MISF, due to technical challenges and reliance on intraoperative imaging.
- Complication rates are generally comparable, though MISF may lead to fewer wound infections and reduced postoperative pain.

### 3. Long-Term Clinical and Functional Outcomes

- Pain relief and functional recovery are similar between MISF and open fusion, with no significant difference in Oswestry Disability Index (ODI) and Visual Analog Scale (VAS) scores at long-term follow-up.
- Fusion rates are comparable, typically exceeding 90% for both techniques with appropriate patient selection and surgical execution.
- Adjacent segment disease (ASD) rates remain inconclusive, with mixed evidence regarding whether MISF provides a protective effect compared to open fusion.

### 4. Cost-Effectiveness and Healthcare Utilization

- MISF has higher initial costs due to specialized instrumentation and surgeon training requirements.
- Long-term cost benefits favor MISF, as reduced complications, shorter hospital stays, and faster return to work may lower overall healthcare expenditures.
- Patient satisfaction is initially higher in MISF, but long-term satisfaction and quality-of-life outcomes appear comparable between the two techniques.

### 5. Future Research and Clinical Implications

- High-quality, long-term randomized controlled trials (RCTs) are needed to better assess fusion durability and ASD progression.
- Advancements in surgical technology, including robotic-assisted fusion and patient-specific implants, may further refine MISF techniques.
- Patient selection remains key—while MISF offers benefits for select cases, open fusion may still be preferable in complex multi-level spinal pathologies.

## Outstanding Questions

1. What are the true long-term differences in clinical outcomes (10+ years) between MISF and traditional open fusion in terms of pain relief, functional recovery, and reoperation rates?
2. Does MISF significantly reduce the risk of adjacent segment disease (ASD), or are the biomechanical advantages overstated in current literature?
3. What patient populations benefit the most from MISF, and in which cases should traditional open fusion still be preferred?
4. How do robotic-assisted and navigation-guided MISF techniques impact fusion success rates, complication risks, and surgical learning curves?
5. Is the higher initial cost of MISF justified by its long-term healthcare savings, and how can cost-effectiveness studies be improved to capture indirect economic benefits?
6. What are the long-term biomechanical effects of MISF compared to open fusion, particularly regarding spinal alignment, load distribution, and muscle preservation?
7. Can advancements in biologics and graft materials further enhance the fusion success rates in MISF and reduce concerns about pseudoarthrosis?
8. What are the best strategies for improving MISF training and standardizing surgical techniques to reduce variability in patient outcomes?
